# Nickel(II), Copper(II) and Zinc(II) Complexes of 9-[2- (Phosphonomethoxy)ethyl]-8-azaadenine (9,8aPMEA), the 8-Aza Derivative of the Antiviral Nucleotide Analogue 9-[2-(Phosphonomethoxy)ethyl] adenine (PMEA). Quantification of Four Isomeric Species in Aqueous Solution

**DOI:** 10.1155/S1565363304000202

**Published:** 2004

**Authors:** Raquel B. Gómez-Coca, Antonín Holý, Rosario A. Vilaplana, Francisco González-Vílchez, Helmut Sigel

**Affiliations:** 1 Department of Chemistry Inorganic Chemistry University of Basel Spitalstrasse 51 Basel CH-4056 Switzerland; 2 Inorganic Chemistry Department Faculty of Chemistry University of Seville Seville E-41071 Spain; 3 Institute of Organic Chemistry and Biochemistry Academy of Sciences Prague CZ-16610 Czech Republic

## Abstract

The acidity constants of the twofold protonated acyclic nucleotide analogue 9-[2-(phosphonomethoxy)- ethyl]-8-azaadenine, H_2_(9,8aPMEA)^±^, as well as the stability constants of the M(H;9,8aPMEA)^+^ and M(9,8aPMEA) complexes with the metal ions M^2+^ =Ni^2+^, Cu^2+^ or Zn^2+^, have been determined by potentiometric pH titrations in aqueous solution at I=0.1 M (NaNO_3_) and 25℃. The result for the release of the first proton from H_2_(9,8aPMEA)^+^ (pK_a_= 2.73), which originates from the (N1)H^+^ site, was confirmed by UV-spectrophotometric measurements. Application of previously determined straight-line plots of log KMM(R-PO_3_) versus PKH_3_(R-HPO_3_)' for simple phosph(on)ate ligands, R- PO-, where R represents a residue without an affinity for metal ions, proves that the primary binding site of 9,8aPMEA^2-^ is the phosphonate group for all three metal ions studied. By stability constant comparisons with related ligands it is shown, in agreement with conclusions reached earlier for the Cu(PMEA) system [PMEA^2-^=dianion of 9-[2- (phosphonomethoxy)ethyl]adenine], that in total four different isomers are in equilibrium with each other, i.e. (i) an open isomer with a sole phosphonate coordination, M(PA)_op_, where PA^2-^=PMEA^2-^or 9,8aPMEA^2-^, (ii) an isomer with a 5-membered chelate involving the ether oxygen, M(PA)cl/o, (iii) an isomer which contains 5- and 7-membered chelates formed by coordination of the phosphonate group, the ether oxygen and the N3 site of the adenine residue, M(PA)_cl/O/N3_, and finally (iv) a macrochelated isomer involving N7, M(PA)_cl/]N7_. The Cu^2+^ systems of PMEA^2-^ and 9,8aPMEA^2-^ behave quite alike; the formation degrees for Cu(PA)_op_, CuM(PA)_cl/O_, Cu(PA)_cl/O/N3_ and Cu(PA)_cl/N3_ are approximately 16, 32, 45 and 7%, respectively, which shows that Cu(PA)_cl/N7_ is a minority species. In the Ni^2+^ and Zn^2+^ systems the open isomer is the dominating one followed by M(PA)_cl/O_, but there are indications that the other two isomers also occur to some extent.


### Nickel(II), Copper(II) and Zinc(II) Complexes of 9-[2-(Phosphonomethoxy)ethyl]-8-azaadenine (9,8aPMEA), the 8-Aza Derivative of the Antiviral Nucleotide Analogue 9-[2-(Phosphonomethoxy)ethyl]adenine (PMEA). Quantification of Four Isomeric Species in Aqueous Solution

Raquel B. Gómez-Coca,[1,2] Antonín Holý,[3] Rosario A. Vilaplana,[2], Francisco González-Vílchez[2] and Helmut Sigel[1,*]

[1] *Department of Chemistry, Inorganic Chemistry, University of Basel, Spitalstrasse 51, CH-4056 Basel, Switzerland*

[2] *Inorganic Chemistry Department, Faculty of Chemistry, University of Seville, E-41071 Seville, Spain*

[3] *Institute of Organic Chemistry and Biochemistry, Academy of Sciences, CZ-16610 Prague, Czech Republic*

(Received: May 23, 2002)

**ABSTRACT**

The acidity constants of the twofold protonated acyclic nucleotide analogue 9-[2-(phosphonomethoxy)-ethyl]-8-azaadenine, $H_2(9,8aPMEA)^{\pm}$, as well as the stability constants of the $M(H;9,8aPMEA)^+$ and M(9,8aPMEA) complexes with the metal ions $M^{2+}$ = $Ni^{2+}$, $Cu^{2+}$ or $Zn^{2+}$, have been determined by potentiometric pH titrations in aqueous solution at $I$ = 0.1 M ($NaNO_3$) and 25°C. The result for the release of the first proton from $H_2(9,8aPMEA)^+$ (p$K_a$ = 2.73), which originates from the $(N1)H^+$ site, was confirmed by UV-spectrophotometric measurements. Application of previously determined straight-line plots of log $K^M_{M(R-PO_3)}$ *versus* p$K^H_{H(R-PO_3)}$, for simple phosph(on)ate ligands, $R-PO_3^{2-}$, where R represents a residue without an affinity for metal ions, proves that the primary binding site of $9,8aPMEA^{2-}$ is the phosphonate group for all three metal ions studied. By stability constant comparisons with related ligands it is shown, in agreement with conclusions reached earlier for the Cu(PMEA) system [$PMEA^{2-}$ = dianion of 9-[2-

* Correspondence should be addressed to

Prof. Dr. Helmut Sigel, Department of Chemistry, Inorganic Chemistry, University of Basel

Spitalstrasse 51, CH-4056 Basel/Switzerland, Fax: ++41-61-267 1017; E-mail: Helmut.Sigel@unibas.ch

(phosphonomethoxy)ethyl]adenine], that in total four different isomers are in equilibrium with each other, i.e. (*i*) an open isomer with a sole phosphonate coordination, $M(PA)_{op}$, where $PA^{2-}$ = $PMEA^{2-}$ or 9,8a$PMEA^{2-}$, (*ii*) an isomer with a 5-membered chelate involving the ether oxygen, $M(PA)_{cl/O}$, (*iii*) an isomer which contains 5- and 7-membered chelates formed by coordination of the phosphonate group, the ether oxygen and the N3 site of the adenine residue, $M(PA)_{cl/O/N3}$, and finally (*iv*) a macrochelated isomer involving N7, $M(PA)_{cl]N7}$. The $Cu^{2+}$ systems of $PMEA^{2-}$ and 9,8a$PMEA^{2-}$ behave quite alike; the formation degrees for $Cu(PA)_{op}$, $CuM(PA)_{cl/O}$, $Cu(PA)_{cl/O/N3}$ and $Cu(PA)_{cl/N7}$ are approximately 16, 32, 45 and 7%, respectively, which shows that $Cu(PA)_{cl/N7}$ is a minority species. In the $Ni^{2+}$ and $Zn^{2+}$ systems the open isomer is the dominating one followed by $M(PA)_{cl/O}$, but there are indications that the other two isomers also occur to some extent.

### 1. INTRODUCTION

The acyclic nucleoside phosphonate, 9-[2-(phosphonomethoxy)ethyl]adenine (PMEA), also known as *Adefovir* [1], can be considered as an analogue of (2'-deoxy)adenosine 5'-monophosphate ((d)$AMP^{2-}$) [2]. PMEA has excellent antiviral properties [1] and in the form of its bis(pivaloyloxymethyl)ester, *Adefovir dipivoxil*, it has recently been approved by the US Food and Drug Administration (FDA) for the treatment [3] of hepatitis B patients; these people suffer from an infection of a DNA virus.

PMEA and its relatives affect the viral reproduction cycle at the stage of DNA synthesis, i.e., they serve in their diphosphorylated form as substrates for polymerases and lead after their incorporation to the termination of the growing nucleic acid chain [1]. Since polymerases depend on the presence of metal ions [4], we have studied over the past few years the metal ion-binding properties of PMEA in detail [2,5,6], and suggested also a mechanism [7] which explains why diphosphorylated PMEA is initially an excellent substrate for nucleic acid polymerases [8,9].

The stability determining binding site of $PMEA^{2-}$ is the phosphonate group; however, biologically important metal ions like $Mg^{2+}$, $Ca^{2+}$, $Mn^{2+}$ and $Zn^{2+}$ are able to interact also with the ether oxygen atom and this gives rise to the following intramolecular equilibrium (1) [2,5,6]:

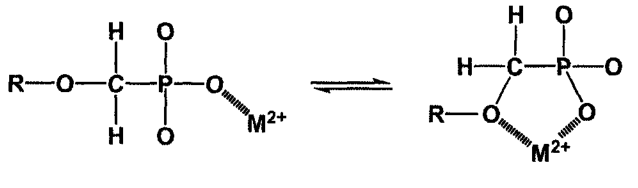

(1)

This proposed metal ion-ether oxygen interaction is crucial for the suggested polymerase mechanism [7] which agrees with the observation that deletion of this ether oxygen or a change in its position in the aliphatic chain leads to compounds which are biologically inactive [8-10].

With certain metal ions like $Cu^{2+}$, $PMEA^{2-}$ may also undergo an adenine interaction. This adenine interaction occurs for a *minority* species via N7 [11], i.e., the phosphonate-coordinated metal ion forms a macrochelate as indicated in equilibrium (2),

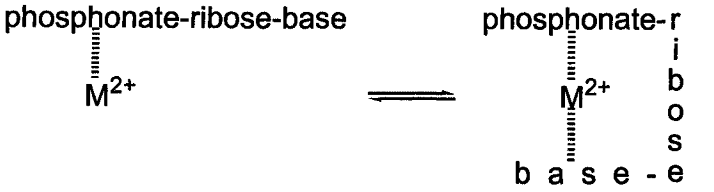

(2)

and which is well known to occur in the complexes of AMP, where a phosphate group is the primary binding site [12,13]. The *majority* species, however, results with $Cu^{2+}$ from an interaction with N3 [2,11,14] in such a way that a M(PMEA) species, which exists as a fivemembered chelate (eq. (1)), forms in addition a seven-membered chelate involving N3; this species is designated as $M(PMEA)_{cl/O/N3}$ and consequently, the macrochelated (eq. (2)) and ether oxygen-bound isomers (eq. (1)) are abbreviated as $M(PMEA)_{cl/N7}$ and $M(PMEA)_{cl/O}$, respectively, and the open isomer seen in equilibria (1) and (2) as $M(PMEA)_{op}$. The indicated situation regarding Cu(PMEA) is most fascinating because for the first time a quantitative evaluation of a system in which four isomers occur in equilibrium was possible [11].

The relative affinities of N3 *versus* N7 of an adenine residue are of general interest since N7 is exposed to the solvent in the major groove of DNA whereas N3 is located in the minor groove [15]. Therefore it was desirable to confirm the observations summarized above for M(PMEA) systems with another acyclic nucleoside phosphonate. We selected 9-[2-(phosphonomethoxy)ethyl]-8-azaadenine (9,8aPMEA) [16], which also exhibits some antiviral activity [17] and which is shown in its dianionic form together with $PMEA^{2-}$ in Figure 1, and studied its metal ion-binding properties with $Ni^{2+}$, $Cu^{2+}$ and $Zn^{2+}$. We selected these metal ions since they are known [18] to have a relatively pronounced affinity toward N donors. To complete the picture, the previously obtained equilibrium data [5,11] for the $Ni^{2+}$ and $Zn^{2+}$ complexes of $PMEA^{2-}$ were now also evaluated regarding the equilibrium scheme (3),

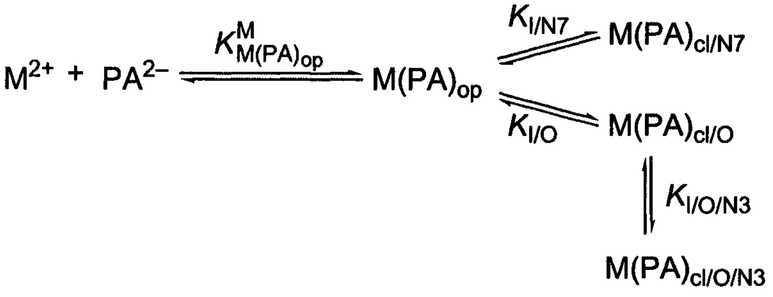

(3)

where $PA^{2-}$ = $PMEA^{2-}$ or $9,8aPMEA^{2-}$. The presented results prove that at least with $Cu^{2+}$ all four isomers occur in solution with both ligands, whereas with $Ni^{2+}$ and $Zn^{2+}$ the proof of their occurrence is more difficult since the differences in complex stability between the various species are small.

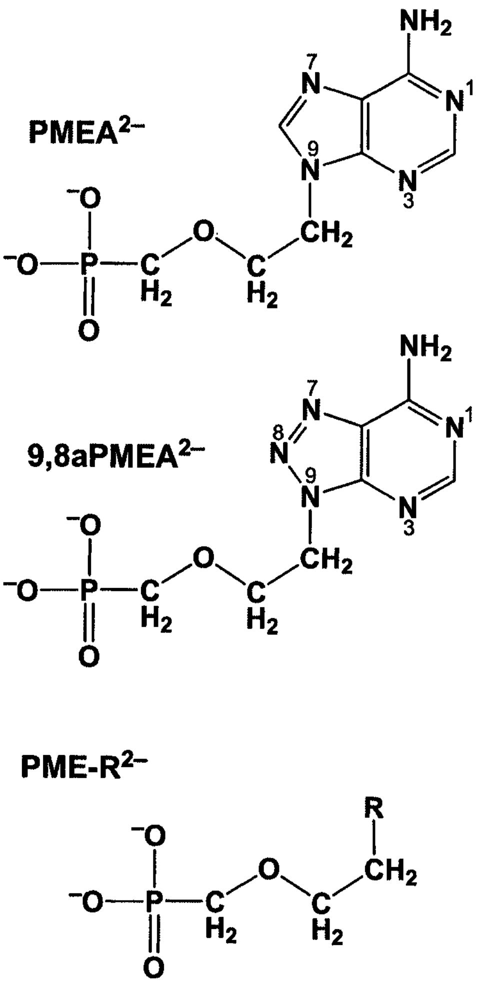

**Fig. 1:** Chemical structures of the dianions of 9-[2-(phosphonomethoxy)ethyl]adenine (= $PMEA^{2-}$ = *Adefovir*) [1] and of 9-[2-(phosphonomethoxy)ethyl]-8-azaadenine (= $9,8aPMEA^{2-}$), together with the structure of $PME\text{-}R^{2-}$, where R is a non-interacting residue, and which represents the metal ion-coordinating properties of the ether-phosphonate chain occurring in $PMEA^{2-}$ and $9,8aPMEA^{2-}$. A further ligand to be considered in this study is 9-(4-phosphonobutyl)adenine, which is abbreviated as $dPMEA^{2-}$ (= 3'-deoxa-$PMEA^{2-}$) to indicate that its structure corresponds to that of $PMEA^{2-}$ except that the ether O atom is replaced by a $CH_2$ group.

### 2. MATERIALS AND METHODS

### 2.1. Materials

Twofold protonated 9-[2-(phosphonomethoxy)ethyl]-8-azaadenine, i.e. $H_2(9,8aPMEA)^{\pm}$, was synthesized by alkylation of 8-azaadenine with a synthon carrying the structural constituents of the required side chain [16]; in fact, the same lot of compound was used as previously [19]. The aqueous stock solutions of the ligand were freshly prepared just before the experiments by dissolving the substance in deionized, ultrapure (MILLI-Q185 PLUS; from Millipore S.A., 67120 Molsheim, France) $CO_2$-free water, adjusted to pH about 8.5 by adding 2 equivalents of 0.1 M NaOH.

The disodium salt of 1,2-diaminoethane-N,N,N',N'-tetraacetic acid ($Na_2H_2EDTA$), potassium hydrogen phthalate, $HNO_3$, NaOH (Titrisol), and the nitrate salts of $Na^+$, $Ni^{2+}$, $Cu^{2+}$ and $Zn^{2+}$ (all *pro analysi*) were from Merck AG, Darmstadt, FRG. All solutions for the potentiometric pH titrations were prepared with ultrapure $CO_2$-free water. The buffer solutions (pH 4.00, 7.00, 9.00 based on the NBS scale; now NIST) used for calibration of the pH-measuring instruments were from Metrohm AG, Herisau, Switzerland.

The exact concentrations of the stock solutions of the divalent metal ions were determined by potentiometric pH titrations *via* their EDTA complexes. The exact concentration of the ligand solutions was in each experiment newly determined by the evaluation of the corresponding titration pairs, i.e. the difference in NaOH consumption between solutions with and without ligand (see Section 2.3).

### 2.2. Potentiometric pH Titrations

The pH titration curves for the determination of the equilibrium constants in $H_2O$ were recorded with a Metrohm E536 potentiograph connected to a Metrohm E665 dosimat and a Metrohm 6.0222.100 combined macro glass electrode. The pH calibration of the instrument was done with the mentioned buffer solutions at pH 4.00, 7.00 and 9.00. The titer of the NaOH used was determined with potassium hydrogen phthalate.

The direct pH meter readings were used in the calculations of the acidity constants; i.e. these constants determined at $I = 0.1$ M ($NaNO_3$) and 25 °C are so-called practical, mixed or Brønsted constants [20]. They may be converted into the corresponding concentration constants by subtracting 0.02 from the listed $pK_a$ values; this conversion term contains both the junction potential of the glass electrode and the hydrogen ion activity [20,21]. It should be emphasized that the ionic product of water ($K_w$) and the mentioned conversion term do not enter into our calculation procedures because we always evaluated the differences in NaOH consumption between a pair of solutions, i.e. with and without ligand. The stability constants determined are, as usual, concentration constants.

All equilibrium constants were calculated by curve-fitting procedures in the way and with the equipment described recently [11, 22].

### 2.3. Determination of Equilibrium Constants

The acidity constants $K^{H}_{H_2(9,8aPMEA)}$ and $K^{H}_{H(9,8aPMEA)}$ of $H_2(9,8aPMEA)^{\pm}$ (see eqs (4) and (5)), where one proton is at the nucleobase moiety and the other at the phosphonate group, were determined by titrating 30 mL of aqueous 2.3-2.5 mM $HNO_3$ (25 °C; $I$ = 0.1 M, $NaNO_3$) in the presence and absence of 0.4 mM deprotonated ligand under $N_2$ with 2.2-2.5 mL of 0.03 M NaOH. The differences in NaOH consumption between such a pair of titrations were used for the calculations. The pH ranges evaluated were 2.8-8.6 and 3.4-7.8. Under these experimental conditions the initial formation degree of $H_2(9,8aPMEA)^{\pm}$ is about 46% and 18%, respectively, and at the end of the titration about 2% and 10% of $H(9,8aPMEA)^-$ are left, respectively. The results for the acidity constants are the averages of 15 pairs of independent titrations.

The stability constants $K^{M}_{M(H;9,8aPMEA)}$ and $K^{M}_{M(9,8aPMEA)}$ of $M(H;9,8aPMEA)^+$ and M(9,8aPMEA) (eqs (6) and (7)), were determined under the same conditions as the acidity constants but now the $HNO_3$ concentration was reduced to 0.83 mM and hence, only 1 mL of 0.03 NaOH was needed for a titration. $NaNO_3$ was partly replaced by $M(NO_3)_2$ (25 °C; $I$ = 0.1 M). The $M^{2+}$/ligand ratios were for $Cu^{2+}$ 11:1 and 5.5:1, for $Ni^{2+}$ 50:1 and 25:1, and for $Zn^{2+}$ 28:1, 26.5:1 and 11:1.

The stability constants were calculated [23] by considering the species $H^+$, $H_2(9,8aPMEA)^{\pm}$, $H(9,8aPMEA)^-$, $9,8aPMEA^{2-}$, $M^{2+}$, $M(H;9,8aPMEA)^+$ and M(9,8aPMEA). The experimental data were collected every 0.1 pH unit from about 4% ($Ni^{2+}$), 1.6% ($Cu^{2+}$) and 2.4% ($Zn^{2+}$) complex formation of $M(H;9,8aPMEA)^+$ to a neutralization degree of about 90% with respect to the species $H(9,8aPMEA)^-$, or until the beginning of the hydrolysis of $M(aq)^{2+}$, which was evident from the titrations without ligand. The maximal formation degrees for the $Ni(H;9,8aPMEA)^+$, $Cu(H;9,8aPMEA)^+$ and $Zn(H;9,8aPMEA)^+$ complexes were only 8.7%, 3.3% and 6.3%, respectively, and hence, the stability constants of the monoprotonated $M(H;9,8aPMEA)^+$ species are estimates only. For the Ni(9,8aPMEA), Cu(9,8aPMEA) and Zn(9,8aPMEA) complexes the maximal formation degree reached in the experiments was about 71%, 51%, and 18%, respectively; the reason for the low formation degree of Zn(9,8aPMEA) is that the experiments were hampered by precipitation.

The individual results for the stability constants showed no dependence on pH or on the excess of metal ion concentration used. The results are in each case the averages of at least 5 independent pairs of titration curves.

### 2.4. Spectrophotometric Measurements

The acidity constant that describes the release of the proton from the $(N1)H^+$ site of the adenine residue in $H_2(9,8aPMEA)^{\pm}$, $pK^{H}_{H_2(9,8aPMEA)}$ (eq (4)), was also determined by spectrophotometry. The UV-Vis spectra of 9,8aPMEA (1.2 mM) were recorded in aqueous solution (25 °C; $I$ = 0.1 M, NaCl) and 1-cm quartz cells with a Varian Cary 3C spectrophotometer connected to an IBM-compatible desk computer (OS/2 system) and an EPSON Stylus 1500 printer. The pH of the solutions was adjusted by dotting with relatively concentrated HCl and measured with a Metrohm 713 pH meter using a Metrohm 6.204.100 glass electrode.

The spectra were recorded within the range of 205 to 330 nm; for further details see Figures 2 and 3 in Section 3.1.

### 3. RESULTS AND DISCUSSION

Derivatives of purines are well known to undergo self-association via π-stacking [24]. Therefore, all potentiometric pH titrations (25 °C; $I$ = 0.1 M, $NaNO_3$), the results of which are summarized below, were carried out with a ligand concentration of 0.4 mM. Under these conditions self-stacking is negligibly small as has been shown for PMEA [5]. Hence, it is ascertained that the results given below reflect the properties of monomeric species.

### 3.1. Acidity Constants of $H_2(9,8aPMEA)^{\pm}$

From the structure of $9,8aPMEA^{2-}$ (see Figure 1) it is evident that this species can accept three protons, two at the phosphonate group and one at the N1 site of the 8-azaadenine residue [25,26]. Further protonations at an adenine residue are possible at N7 and N3, but these protons are released with $pK_a < 0$ [27]; similarly, release of the first proton from the $-P(O)(OH)_2$ group of $H_3(PMEA)^+$ occurs with $pK_a$ = 1.2 [26,28] and the same may be surmised for $H_3(9,8aPMEA)^{\pm}$. Hence, in the present study, for which all potentiometric pH titrations were carried out at pH ≥ 2.8, only the following two deprotonation reactions, in which $9,8aPMEA^{2-}$ and related species like $PMEA^{2-}$ (Figure 1) are abbreviated as $PA^{2-}$ (this also holds for other equations further below), need to be considered:

$$H_2(PA)^{\pm} \rightleftharpoons H(PA)^- + H^+ \tag{4a}$$

$$K^H_{H_2(PA)} = [H(PA)^-][H^+]/[H_2(PA)^{\pm}] \tag{4b}$$

$$H(PA)^- \rightleftharpoons PA^{2-} + H^+ \tag{5a}$$

$$K^H_{H(PA)} = [PA^{2-}][H^+]/[H(PA)^-] \tag{5b}$$

Indeed, all the experimental data from the potentiometric pH titrations in aqueous solution could be excellently fitted by taking into account equilibria (4) and (5). The acidity constants obtained in the present study for $H_2(9,8aPMEA)^{\pm}$ are given in Table 1 together with some related data [29-31].

From a quick comparison of the acidity constants in Table 1 it is immediately evident that the first proton released from $H_2(9,8aPMEA)^{\pm}$ according to equilibrium (4) is from the $(N1)H^+$ site and the second one according to equilibrium (5) from the $-P(O)_2(OH)^-$ group. This site attribution is confirmed by the spectrophotometric measurements seen in Figure 2; the change in absorption of the $H_2(9,8aPMEA)^{\pm}$/

**Table 1**

Negative Logarithms of the Acidity Constants of $H_2(9,8aPMEA)^{\pm}$ and $H_2(PMEA)^{\pm}$ (eqs (4) and (5)), as Determined by Potentiometric pH Titrations in Aqueous Solution (25 °C; $I$ = 0.1 M, $NaNO_3$), Together with Some Further Related Data[a]

| No | Protonated species | $pK^H_{H_2(PA)}$ $(N1)H^+$ | $pK^H_{H(PA)}$ $P(O)_2(OH)^-$ | Ref. |
|---|---|---|---|---|
| 1 | $H(9Me8azaAde)^+$ | 2.70/2.80 | | [25,29][b] |
| 2 | $H_2(9,8aPMEA)^{\pm}$ | 2.73 ± 0.02 | 6.85 ± 0.02 | –[c] |
| 3 | $H_2(PMEA)^{\pm}$ | 4.16 ± 0.02 | 6.90 ± 0.01 | [5,26] |
| 4 | $H(PME\text{-}R)^-$ | | 6.99 ± 0.04[d] | [5,30] |
| 5 | $H_2(dPMEA)^{\pm}$ | 4.17 ± 0.02 | 7.69 ± 0.01 | [11] |
| 6 | $CH_3P(O)_2(OH)^-$ | | 7.51 ± 0.01 | [31] |

[a] The error limits given are *three times* the standard error of the mean value or the sum of the probable systematic errors, whichever is larger. So-called practical (or mixed) acidity constants are listed; see Section 2.2.

[b] Determined by $^1$H-NMR shift [25] and spectrophotometric [29] measurements, respectively; 9Me8azaAde = 9-methyl-8-azaadenine.

[c] The result $pK^H_{H_2(9,8aPMEA)} = 2.73 \pm 0.02$ was confirmed by spectrophotometric measurements (see Figures 2 and 3); $pK^H_{H_2(9,8aPMEA)} = 2.73 \pm 0.08$.[a]

[d] Average value from compounds like $R\text{-}CH_2CH_2\text{-}O\text{-}CH_2\text{-}P(O)_2(OH)^-$, where R = H or cytosine (bound via N1); for details see ref. [30].

$H(9,8aPMEA)^-$ pair occurs in this range of wavelengths where protonation/deprotonation reactions of related aromatic moieties are commonly seen [32].

A further reason for the spectrophotometric measurements was that the formation degree of the $H_2(9,8aPMEA)^{\pm}$ species that could be reached in the potentiometric pH titrations was relatively low (see Section 2.3). This means that it was desirable to determine the acidity constant for equilibrium (4) also by another independent method. Therefore we measured the absorption spectra of 9,8aPMEA as a function of pH; a representative set of spectra is shown in Figure 2. The evaluation of the same experiment by a curve-fitting procedure, but involving more data, is given in Figure 3. Since $NaNO_3$ absorbs in part of the wavelength range needed for the evaluation of 9,8aPMEA data, $I$ was now adjusted to 0.1 M with NaCl. The final result from two independent series of measurements is $pK^H_{H_2(9,8aPMEA)}$ = 2.73 ± 0.08, and this value is in excellent agreement with the constant given in Table 1 and determined by potentiometry.

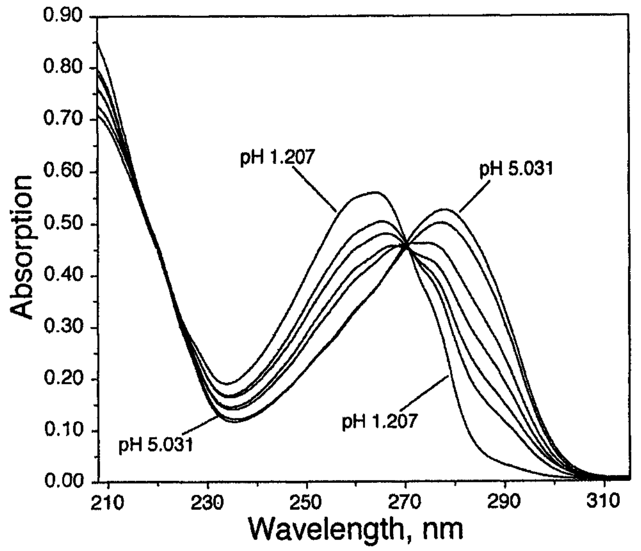

**Fig. 2:** UV absorption spectra measured in 1-cm quartz cells of 9,8aPMEA (1.2 mM) in aqueous solution in dependence on pH; i.e., the pH values varied from 1.207, 2.286, 2.525, 2.796, 3.047, 3.841 to 5.031. The sample beam contained 9,8aPMEA, HCl and NaCl, and the reference beam HCl and NaCl (25 °C; $I$ = 0.1 M, NaCl). For the evaluation of the spectra see Figure 3.

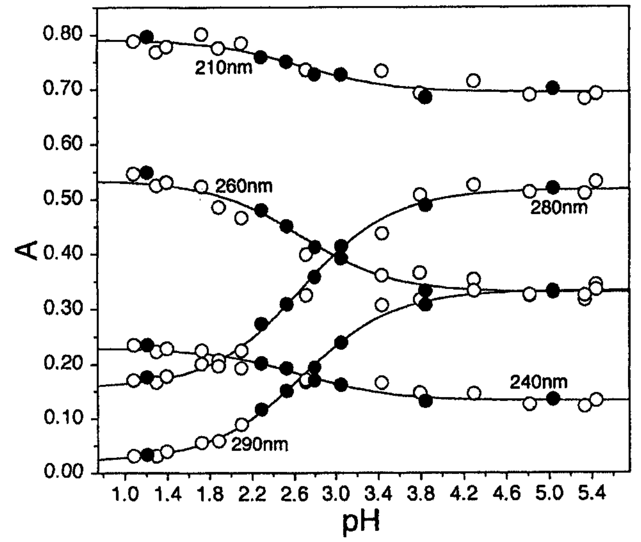

**Fig. 3:** The UV absorption spectra of 9,8aPMEA (Figure 2) in aqueous solution were evaluated at 210, 240, 260, 280 and 290 nm in dependence on pH. These evaluations furnished only the first acidity constant of $H_2(9,8aPMEA)^{\pm}$. Giving the averaged result (weighted mean) $pK^H_{H_2(9,8a,PMEA)}$ = 2.67 ± 0.10 (3σ) for this experiment (25 °C; $I$ = 0.1 M, NaCl). The solid curves shown are the computer calculated best fits for the various wavelengths through the experimental data points obtained at pH 1.082, 1.207, 1.294, 1.389, 1.719, 1.881, 2.095, 2.286, 2.525, 2.712, 2.796, 3.047, 3.432, 3.788, 3.841, 4.291, 4.811, 5.031, 5.331 and 5.436 (from left to right) by using the mentioned average of the acidity constant. The seven solid (•) points, i.e., at pH 1.207, 2.286, 2.525, 2.796, 3.047, 3.841 and 5.031 are those that correspond to the spectra shown in Figure 2. The final result ($pK^H_{H_2(9,8aPMEA)}$ = 2.73 ± 0.08 (3σ)) is the average of two independent experimental series.

The most obvious conclusions from the data in Table 1 are that replacement of (C8)H by a nitrogen atom reduces the $pK_a$ of the $(N1)H^+$ site by about $\Delta pK_a = 1.5$, i.e., this site becomes considerably more acidic as follows from a comparison of entries 1 and 2 with 3 and 5. In contrast, entries 2-4 demonstrate that the nucleobase residue hardly affects the release of the proton from the $-P(O)_2(OH)^-$ group. However, elimination of the ether oxygen from the $R-CH_2CH_2-O-CH_2-PO_3^{2-}$ chain enhances the basicity of the $-PO_3^{2-}$ group remarkably (cf. entries 2-6).

### 3.2. Stability Constants of the $M(H;9,8aPMEA)^+$ and M(9,8aPMEA) Complexes

Since under the experimental conditions the metal ions ($M^{2+}$) are present in a large excess compared to the concentration of the ligand only the following two equilibria need to be considered for complex formation:

$$M^{2+} + H(PA)^- \rightleftharpoons M(H;PA)^+ \tag{6a}$$

$$K^{M}_{M(H;PA)} = [M(H;PA)^+]/([M^{2+}][H(PA)^-]) \tag{6b}$$

$$M^{2+} + PA^{2-} \rightleftharpoons M(PA) \tag{7a}$$

$$K^{M}_{M(PA)} = [M(PA)]/([M^{2+}][PA^{2-}]) \tag{7b}$$

It should be noted that in formulas like $M(H;PA)^+$ the $H^+$ and $PA^{2-}$ are separated by a semicolon to facilitate reading, yet they appear within the same parentheses to indicate that the proton is at the ligand without defining its location.

Indeed, together with equilibria (4) and (5), equilibria (6) and (7) are sufficient to obtain excellent fitting of the titration data (see Section 2.3), provided the evaluation is not carried into the pH range where formation of hydroxo species occurs, which was evident from the titrations without ligand. Of course, equilibria (6) and (7) are also connected via equilibrium (8)

$$M(H;PA)^+ \rightleftharpoons M(PA) + H^+ \tag{8a}$$

$$K^{H}_{M(H;PA)} = [M(PA)][H^+]/[M(H;PA)^+] \tag{8b}$$

and the corresponding acidity constant (eq. (8b)) may be calculated with equation (9) [33]:

$$pK^{H}_{M(H;PA)} = pK^{H}_{H(PA)} + \log K^{M}_{M(H;PA)} - \log K^{M}_{M(PA)} \tag{9}$$

The results are listed in column 4 of Table 2 together with the constants for the corresponding M(PMEA) complexes and some further related data. The stability constants given in footnote "e" for the

$M(H;9,8aPMEA)^{+}$ complexes need to be considered as estimates since the formation degree of these species was low (see Section 2.3). The stability constants of the M(9,8aPMEA) complexes show the trend expected for divalent 3d metal ions, i.e., they vary within the series $Ni^{2+} < Cu^{2+} > Zn^{2+}$, and this holds for the constants due to the $M(H;9,8aPMEA)^{+}$ species as well.

The analysis of potentiometric pH titrations only yields the amount and distribution of the species of a net charged type; i.e., further information is required to locate the binding sites of the proton and the metal ion in the $M(H;9,8aPMEA)^{+}$ species. At first one may ask where the proton is located because binding of a metal ion to a protonated ligand commonly leads to an acidification of the ligand-bound proton [34,35]. Hence, the acidity constants according to equilibrium (8) are needed; these values are calculated with the data listed in Tables 1 and 2 by application of equation (9) to give the following results:

$$pK^{H}_{Ni(H;9,8aPMEA)} = 5.30 \pm 0.26 \quad (10a)$$

$$pK^{H}_{Cu(H;9,8aPMEA)} = 3.82 \pm 0.25 \quad (10b)$$

$$pK^{H}_{Zn(H;9,8aPMEA)} = 4.83 \pm 0.27 \quad (10c)$$

It is revealing to see that these acidity constants of the $M(H;9,8aPMEA)^{+}$ complexes are by about 1.5 to 3.0 log units smaller than $pK^{H}_{H(H;9,8aPMEA)} = 6.85 \pm 0.02$ (Table 1) but approximately 1.1 to 2.6 log units larger than $pK^{H}_{H_2(H;9,8aPMEA)} = 2.73 \pm 0.02$ (Table 1). This comparison shows that the proton in $M(H;9,8aPMEA)^{+}$ is bound to the phosphonate group, hence, one may tentatively assume that the metal ion is coordinated preferentially to the nucleobase, since a monoprotonated phosphonate group is only a weak binding site. Indeed, this suggestion agrees with evidence obtained previously for other related $M(H;PA)^{+}$ species [5,14,36].

### 3.3. Evaluation of the Stabilities of the M(9,8aPMEA) Complexes

For the M(9,8aPMEA) complexes the question arises: Does the 8-azaadenine residue also participate in metal ion binding next to the phosphonate group? Should such an additional interaction with the nucleobase residue occur then it has to be reflected in an increased complex stability [37]. Hence, it is necessary to define the stability of a pure $-PO_3^{2-} / M^{2+}$ interaction. This can be done by applying the previously defined [5] straight-line correlations which are based on log $K^{M}_{M(R-PO_3)}$ *versus* $pK^{H}_{H(R-PO_3)}$ plots for simple phosphate monoesters [38] and phosphonates [5]; these ligands are abbreviated as $R-PO_3^{2-}$, where R represents a noncoordinating residue. The parameters for the corresponding straight-line equations, which are defined by equation (11),

$$\log K^{M}_{M(R-PO_3)} = m \cdot pK^{H}_{H(R-PO_3)} + b \quad (11)$$

have been tabulated [2a,5,39,40], i.e., the slopes *m* and the intercepts *b* with the *y*-axis. Hence, with a known $pK_a$ value for the deprotonation of a $-P(O)_2(OH)^-$ group an expected stability constant can be calculated for any phosph(on)ate-metal ion complex.

The plots of log $K^{M}_{M(R-PO_3)}$ *versus* $pK^{H}_{H(R-PO_3)}$ according to equation (11) are shown in Figure 4 for the 1:1 complexes of $Cu^{2+}$ and $Zn^{2+}$, as examples, with the data points (empty circles) of the eight simple ligand systems used [5] for the determination of the straight baselines. The two solid circles refer to the corresponding M(9,8aPMEA) complexes and the crossed ones to the M(PMEA) species. For further comparison also the data points for the related M(PME-R) (solid squares) and M(dPMEA) (empty squares) systems are shown.

All the latter mentioned data points are clearly positioned above their reference lines thus proving that beyond the $-PO_3^{2-}/M^{2+}$ binding additional interactions occur. The smallest stability increase is observed for the M(dPMEA) complexes, where $dPMEA^{2-}$ = 3'-deoxa-$PMEA^{2-}$ (i.e., the ether O is replaced by $CH_2$) = 9-(4-phosphonobutyl)adenine (Figure 1); in these instances macrochelates according to equilibrium (2) involving N7 of the adenine residue are formed [11]. For the M(PME-R) complexes the stability increase is more pronounced and clearly attributable to equilibrium (1) since no other additional binding site but the ether O atom is available (Figure 1) [5,30]. However, the stability increase observed for the Cu(9,8aPMEA), Cu(PMEA) and Zn(9,8aPMEA) species is much larger than the one for the M(dPMEA) and M(PME-R) complexes, thus indicating that an accumulation of extra interactions occurs as it is depicted in the equilibrium scheme (3). No meaning should be attributed to the apparent equality of the stability increase seen in Figure 4 for the Zn(PMEA) and Zn(PME-R) complexes because the stability constant for Zn(PMEA) is only an estimate carrying a large error limit (see Table 2, entry 1c in column 4).

### 3.4. Extent of the Total Amount of Chelates Formed in the M(PA) Systems

Before considering the situation in the M(PMEA) and M(9,8aPMEA) complexes according to the equilibrium scheme (3) in more detail (see Section 3.5), it is appropriate to evaluate first the total amount of closed species, $M(PA)_{cl/tot}$, for all four $PA^{2-}$ ligands considered (Figure 1) because evidently the sum of all the closed species, independent of their structure, is responsible for the observed stability increase. Stability enhancements like those seen in Figure 4 can be quantified by the differences between the experimentally (exptl) measured stability constants and those calculated (calcd) according to equation (11); this difference is defined in equation (12),

$$\log \Delta_{M/PA} = \log K^{M}_{M(PA)_{exptl}} - \log K^{M}_{M(PA)_{calcd}} \quad (12a)$$

$$= \log K^{M}_{M(PA)} - \log K^{M}_{M(PA)_{op}} \quad (12b)$$

where the expressions $\log K^{M}_{M(PA)_{calcd}}$ and log $K^{M}_{M(PA)_{op}}$ are synonymous because the calculated value equals the stability constant of the 'open' isomer, $M(PA)_{op}$ (see equilibria (1)-(3)), in which only a $-PO_3^{2-}/M^{2+}$ interaction occurs. In columns 4-6 of Table 2 the values for the terms of equation (12) are listed.

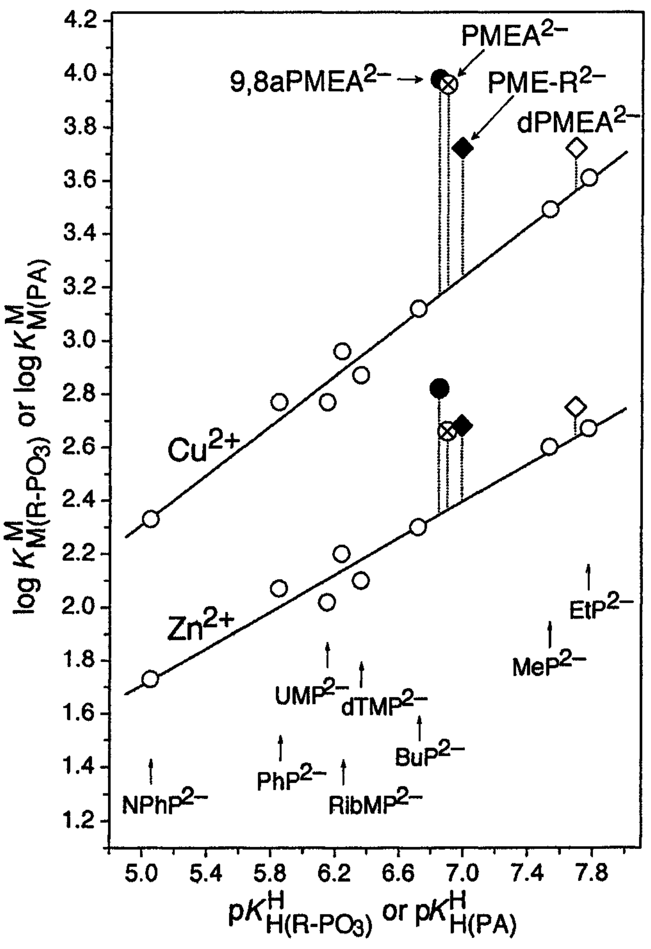

**Fig. 4:** Evidence for an enhanced stability of the M(PMEA) (⊗) and M(9,8aPMEA) (•) complexes of $Cu^{2+}$ and $Zn^{2+}$ in comparison with the stability of the corresponding complexes formed with PME-$R^{2-}$ (◆) and dPMEA$^{2-}$ (◇) (for the structures of the $PA^{2-}$ ligands see Figure 1), based on the relationship between log $K^{M}_{M(R-PO_3)}$ *versus* p$K^{H}_{H(R-PO_3)}$ for M(R-$PO_3$) complexes of simple phosphate monoester and phosphonate ligands (R-$PO_3^{2-}$) (O): 4-nitrophenyl phosphate (NPhP$^{2-}$), phenyl phosphate (PhP$^{2-}$), uridine 5'-monophosphate (UMP$^{2-}$), D-ribose 5-monophosphate (RibMP$^{2-}$), thymidine [= 1-(2-deoxy-β-D-ribofuranosyl)thymine] 5'-monophosphate (dTMP$^{2-}$), *n*-butyl phosphate (BuP$^{2-}$), methanephosphonate (MeP$^{2-}$) and ethanephosphonate (EtP$^{2-}$) (from left to right). The least-squares lines (eq. (11)) are drawn through the corresponding 8 data sets (O) taken from ref. [38] for the phosphate monoesters and from ref. [5] for the phosphonates. The points due to the equilibrium constants for the $M^{2+}/PA^{2-}$ systems are based on the values listed in Tables 1 (column 4) and 2 (columns 4 or 6). The vertical broken lines emphasize the stability differences from the reference lines; they equal log $\Delta_{M/PA}$ as defined in eq. (12) for the M(PA) complexes. All the plotted equilibrium constants refer to aqueous solutions at 25 °C and $I$ = 0.1 M ($NaNO_3$).

**Table 2**

All values for log $\Delta_{M/PA}$ are positive with the single exception of the one for the Zn(dPMEA) complex where log $\Delta_{Zn/dPMEA}$ is zero within the error limits (Table 2, entry 4c in column 6).

The 'total' of the dimensionless intramolecular equilibrium constant, $K_{I/tot}$, is defined by equation (13) (see also below eq. (21)),

$$K_{I/tot} = [M(PA)_{cl/tot}/][M(PA)_{op}] \quad (13)$$

and values for $K_{I/tot}$ can be calculated following known procedures [5,12,37,39,40], i.e., via equation (14):

$$K_{I/tot} = 10^{\log \Delta_{M/PA}} - 1 \quad (14)$$

Knowledge of $K_{I/tot}$ allows then according to equation (15)

$$\% \ M(PA)_{cl/tot} = 100 \cdot K_{I/tot}](1 + K_{I/tot}) \quad (15)$$

to obtain the percentage of the sum of all the closed isomers (cl/tot) present in equilibrium, i.e., their total formation degree. The corresponding results for the four $PA^{2-}$ ligands of Figure 1 and their $Ni^{2+}$, $Cu^{2+}$ and $Zn^{2+}$ complexes are summarized in columns 6-8 of Table 2.

The most easily understood result of the evaluation is the one given under entry 3 in Table 2 because the PME-$R^{2-}$ ligand can only form the two isomeric complexes seen in equilibrium (1), i.e. here only the open species, $M(PA)_{op}$, and the ether oxygen-closed one, $M(PA)_{cl/O}$, exist and therefore in these cases $K_{I/tot} = K_{I/O}$ (Table 2, column 7), which is defined by equation (16),

$$K_{I/O} = [M(PA)_{cl/O/}][M(PA)_{op}] \quad (16)$$

and % $M(PA)_{cl/tot}$ = % $M(PME\text{-}R)_{cl/O}$ (Table 2, column 8). Similarly simple is the situation with $dPMEA^{2-}$ because in this case an additional metal ion interaction, next to the one with the $-PO_3^{2-}$ group, must occur with the adenine residue and it was previously concluded [11] that this is the N7 site; hence, here equilibrium (2) applies. Consequently, for the M(dPMEA) complexes it holds $K_{I/tot} = K_{I/N7}$, as defined by equation (17),

$$K_{I/N7} = [M(PA)_{cl/N7}]/[M(PA)_{op}] \quad (17)$$

and % $M(PA)_{cl/tot}$ = % $M(dPMEA)_{cl/N7}$ (Table 2, columns 7 and 8).

It is evident that the situation for the complexes formed with $PMEA^{2-}$ and 9,8a$PMEA^{2-}$ is more complicated, since more possibilities for the formation of closed isomers exist, and that these possibilities materialize at least in part is evident from the observed rather large stability increases, log $\Delta_{M/PA}$ (Table 2, column 6), and also from the high formation degrees calculated for % $M(PA)_{cl/tot}$. Furthermore, it is revealing to see that the values given in column 8 of Table 2 for % $M(PMEA)_{cl/tot}$ and % $M(9,8aPMEA)_{cl/tot}$ (entries 1 and 2) are for a given metal ion very similar or even identical within their error limits.

### 3.5. Formation Degrees of the Four Isomers Existing in Equilibrium for the M(PMEA) and M(9,8aPMEA) Species

Up to now the $Cu^{2+}$/PMEA system is the one most thoroughly studied. Indeed, it had originally been proven [14] that three isomers are important for the Cu(PMEA) system [7]: (*i*) An 'open' isomer, $Cu(PMEA)_{op}$, in which the metal ion is solely coordinated to the phosphonate group; (*ii*) an isomer which involves the ether oxygen (see Figure 1) as shown in equilibrium (1), designated as $Cu(PMEA)_{cl/O}$; and (*iii*) an isomer in which not only a 5-membered chelate but in addition a 7-membered one involving N3 exists, i.e. $Cu(PMEA)_{cl/O/N3}$. More recently [11] evidence was provided that there is a fourth isomer, a minority species, in which the phosphonate-coordinated $Cu^{2+}$ interacts with N7 of the adenine residue forming a macrochelate, $Cu(PMEA)_{cl/N7}$, as indicated in equilibrium (2). In this context it is important to emphasize that for steric reasons no macrochelate involving *only* N3 can be formed by $PMEA^{2-}$ and $Cu^{2+}$ [2a]. If one tries to form such a species with molecular models, one automatically forces the ether oxygen into the coordination sphere of the metal ion, giving rise to the already mentioned $Cu(PMEA)_{cl/O/N3}$ isomer [2a]. If one summarizes all these results then the simple equilibrium (7a) must be replaced for the Cu(PMEA) system by the rather complicated equilibrium scheme (3) already introduced in Section 1. Of course, exactly the same reasonings also apply to the $PMEA^{2-}$ complexes formed with $Ni^{2+}$ and $Zn^{2+}$ as well as for the M(9,8aPMEA) species. For these systems a quantitative evaluation toward the formation degree of the various isomers needs now to be carried out.

The four equilibrium constants seen in scheme (3) are defined by the already mentioned equations (16) and (17) together with the also necessary equations (18) and (19):

$$K^{M}_{M(PA)_{op}} = [M(PA)_{op}]/([M^{2+}][PA^{2-}]) \qquad (18)$$

$$K_{I/O/N3} = [M(PA)_{cl/O/N3}]/[M(PA)_{cl/O}] \qquad (19)$$

With these definitions the measured overall stability constant (eq. (7b)) can be redefined as given in equations (20a)-(20d):

$$K^{M}_{M(PA)} = \frac{[M(PA)]}{[M^{2+}][PA^{2-}]} \qquad (20a)$$

$$= \frac{[M(PA)_{op}]+[M(PA)_{cl/N7}]+[M(PA)_{cl/O}]+[M(PA)_{cl/O/N3}]}{[M^{2+}][PA^{2-}]} \qquad (20b)$$

$$= K^{M}_{M(PA)_{op}} + K_{I/N7}\bullet K^{M}_{M(PA)_{op}} + K_{I/O}\bullet K^{M}_{M(PA)_{op}} + K_{I/O/N3}\bullet K_{I/O}\bullet K^{M}_{M(PA)_{op}} \qquad (20c)$$

$$= K^{M}_{M(PA)_{op}}(1 + K_{I/N7} + K_{I/O} + K_{I/O}\bullet K_{I/O/N3}) \qquad (20d)$$

The connection between the overall intramolecular equilibrium constant $K_{I/tot}$, already introduced in Section 3.4, and the accessible stability enhancement (eq. (12)) is given by equations (21a) - (21e):

$$K_{I/tot} = \frac{K^{M}_{M(PA)}}{K^{M}_{M(PA)_{op}}} - 1 = 10^{\log \Delta_{M/PA}} - 1 \tag{21a}$$

$$= \frac{[M(PA)_{cl/tot}]}{[M(PA)_{op}]} \tag{21b}$$

$$= \frac{[M(PA)_{cl/N7}] + [M(PA)_{cl/O}] + [M(PA)_{cl/O/N3}]}{[M(PA)_{op}]} \tag{21c}$$

$$= K_{I/N7} + K_{I/O} + K_{I/O/N3} \bullet K_{I/O} \tag{21d}$$

$$= K_{I/N7} + K_{I/O}(1 + K_{I/O/N3}) \tag{21e}$$

Values for $K_{I/tot}$ were already calculated with equations (12) and (14) in Section 3.4; they are listed in column 7 of Table 2 (entries 1 and 2). The relation between $K_{I/tot}$ and the other three intramolecular equilibrium constants follows from equations (21b) and (21c). Based on the reasonable assumption [7] that the stability of the $M(PA)_{cl/O}$ isomer, where $PA^{2-} = PMEA^{2-}$ or $9,8aPMEA^{2-}$, is well represented by that of the 5-membered $M(PME\text{-}R)_{cl/O}$ species (Figure 1) and the stability of the $M(PA)_{cl/N7}$ isomer by that of the $M(dPMEA)_{cl/N7}$ macrochelate, values for $K_{I/O}$, which define the position of equilibrium (1), and $K_{I/N7}$, which refer to equilibrium (2), are also known (see the second to the last paragraph in Section 3.4). Hence, the only unknown constant in equation (21e) is $K_{I/O/N3}$ (eq. (19)) and thus values for this constant can be obtained, and consequently, the formation degrees for all four isomers appearing in scheme (3) can now be calculated. The corresponding results are summarized in Table 3 for the M(PMEA) and M(9,8aPMEA) systems; as far as the error limits are concerned it needs to be emphasized that *three times* the standard errors ($3\sigma$) are given.

From Table 3 it is evident that Cu(PMEA) and Cu(9,8aPMEA) (entries 1b and 2b) have practically identical properties: The $Cu(PA)_{cl/O/N3}$ species with the 5- and 7-membered chelate rings dominate with formation degrees of about 45% followed by $Cu(PA)_{cl/O}$ with about 30%. As far as $Cu(PMEA)_{cl/O/N3}$ is concerned, the result with 41 ± 12% is within the error limit identical with the previously obtained 49 ± 10% where the formation of the fourth isomer, $Cu(PMEA)_{cl/N7}$, had not been taken into account [5,7]. This demonstrates immediately that the $Cu(PA)_{cl/N7}$ isomer must be a minority species; indeed, the present calculations show that the formation degrees of $Cu(PMEA)_{cl/N7}$ and $Cu(9,8aPMEA)_{cl/N7}$ amount only to about 7% (see also ref. [11]).

It is interesting to see that for the Ni(PMEA) and Zn(PMEA) systems about 50% each exist as the open isomer and the remaining half of the species is present as chelates (Table 3, entries 1a and 1c). In the case of Ni(PMEA) all three chelated isomers occur with comparable concentrations though the formation degrees of $Ni(PMEA)_{cl/O}$ and $Ni(PMEA)_{cl/O/N3}$ appear to be slightly favored. With Zn(PMEA) the $Zn(PMEA)_{cl/O}$ isomer seems to be the dominating species, the formation degrees of the other chelates being zero within the error

**Table 3**

limits; here it should be recalled that the overall stability constant for Zn(PMEA) is an estimate only (Table 2, entry 1c) [5].

For Zn(9,8aPMEA) (Table 3; entry 2c) the results are more clear-cut since in this case the overall stability constant of the complex could actually be measured (see Section 2.3): Again the $Zn(9,8aPMEA)_{cl/O}$ chelate dominates. However, in this case it may be helpful to rewrite the results for $Zn(9,8aPMEA)_{cl/O}$, $Zn(9,8aPMEA)_{cl/N7}$ and $Zn(9,8aPMEA)_{cl/O/N3}$ with *one* standard deviation ($1\sigma$) only, that is $32 \pm 4$, $10 \pm 7$, and $24 \pm 9\%$, respectively. This view confirms that $Zn(9,8aPMEA)_{cl/O}$ dominates but that $Zn(9,8aPMEA)_{cl/O/N7}$ most likely also exists, whereas $Zn(9,8aPMEA)_{cl/N7}$ is definitely also for this system a minority species. The great similarity between the Zn(PMEA) and Zn(9,8aPMEA) systems is evident, despite all shortcomings, from a comparison of the values in entries 1c and 2c of Table 3. This is also true for the Ni(PMEA) and Ni(9,8aPMEA) systems for which the values seen in entries 1a and 2a of Table 3 overlap within their error limits.

### 4. CONCLUSIONS

The presented results prove that systems in which four different isomers occur in equilibrium in solution can be treated in a quantitative way. They prove further that both N3 and N7 of an adenine residue may bind to metal ions provided primary binding sites promoting a favorable steric orientation are available. With regard to nucleic acids this result is of relevance; in fact, that the more basic N7 [27] is suited for such purposes is by now general knowledge [12,39] whereas this property of N3 has only been recognized more recently [14,27b,35,36a,41].

Furthermore, it is astonishing to see how similar the coordinating properties of the two nucleotide analogues $PMEA^{2-}$ and $9,8aPMEA^{2-}$ (Figure 1) are towards $Ni^{2+}$, $Cu^{2+}$ and $Zn^{2+}$. On the other hand, this observation complements the fact that both acyclic-nucleoside phosphonate analogues exhibit antiviral activity [1,16,17]. Therefore, it is interesting to note that the coordination chemistry of 8-[2-(phosphonomethoxy)ethyl]adenine ($8,8aPMEA^{2-}$) differs [42] from the one described herein, and that indeed this nucleotide analogue does not show any useful biological activity [16,17].

### ACKNOWLEDGEMENTS

The competent technical assistance of Mrs. Rita Baumbusch and Mrs. Astrid Sigel in the preparation of this manuscript, the help of Dr. Larisa E. Kapinos with the spectrophotometric experiments, and stimulating discussions with members of the COST D20 programme are gratefully acknowledged. This study was supported by the Swiss National Science Foundation (H.S.) and the Programme of Targeted Projects (S4055109) of the Academy of Sciences of the Czech Republic (A.H.) as well as within the COST D20 programme by the Swiss Federal Office for Education and Science (H.S.) and the Ministry of Education of the Czech Republic (D.20.002; A.H.). This study also received support from the University of Basel and it is further part of a research project (No. 4055905) of the Institute of Organic Chemistry and Biochemistry (IOCB) in Prague.

### REFERENCES

1. A. Holý, J. Günter, H. Dvořáková, M. Masojídková, G. Andrei, R. Snoeck, J. Balzarini and E. De Clercq, *J. Med. Chem.*, **42**, 2064-2086 (1999) (and refs therein).

2. (a) H. Sigel, *Coord. Chem. Rev.*, **144**, 287-319 (1995). (b) H. Sigel, *J. Indian Chem. Soc.*, **74**, 261-271 (1997) (P. Ray Award Lecture).

3. *Chemische Rundschau* (CH-4501 Solothurn, Switzerland), No. 19; Oct. 8, 2002; p. 68.

4. (a) A. S. Mildvan, *Magnesium*, **6**, 28-33 (1987). (b) C. Klevickis and C. M. Grisham, *Met. Ions Biol. Syst.*, **32**, 1-26 (1996). (c) J. D. Crowley, D. A. Traynor and D. C. Weatherburn, *Met. Ions Biol. Syst.*, **37**, 209-278 (2000).

5. H. Sigel, D. Chen, N. A. Corfù, F. Gregáň, A. Holý and M. Strašák, *Helv. Chim. Acta*, **75**, 2634-2656 (1992).

6. (a) D. Chen, M. Bastian, F. Gregáň, A. Holý and H. Sigel, *J. Chem. Soc., Dalton Trans.*, 1537-1546 (1993). (b) D. Chen, F. Gregáň, A. Holý and H. Sigel, *Inorg. Chem.*, **32**, 5377-5384 (1993). (c) H. Sigel, C. A. Blindauer, A. Holý and H. Dvořáková, *Chem. Commun.*, 1219-1220 (1998). (d) C. A. Blindauer, A. Holý, H. Dvořáková and H. Sigel, *J. Biol. Inorg. Chem.*, **3**, 423-433 (1998). (e) G. Kampf, M. S. Lüth, L. E. Kapinos, J. Müller, A. Holý, B. Lippert and H. Sigel, *Chem. Eur. J.*, **7**, 1899-1908 (2001). (f) R. B. Gómez-Coca, L. E. Kapinos, A. Holý, R. A. Vilaplana, F. González-Vílchez and H. Sigel, *J. Inorg. Biochem.*, **84**, 39-46 (2001).

7. (a) H. Sigel, *Pure Appl. Chem.*, **71**, 1727-1740 (1999). (b) H. Sigel, *Chem. Soc. Rev.*, **33**, 191-200 (2004).

8. A. Holý, E. De Clercq and I. Votruba, *ACS Symp. Ser.*, **401**, 51-71 (1989).

9. A. Holý, I. Votruba, A. Merta, J. Černý, J. Veselý, J. Vlach, K. Šedivá, I. Rosenberg, M. Otmar, H. Hřebabecký, M. Trávníček, V. Vonka, R. Snoeck and E. De Clercq, *Antiviral Res.*, **13**, 295-311 (1990).

10. D. Villemin and F. Thibault-Starzyk, *Synth. Commun.*, **23**, 1053-1059 (1993).

11. R. B. Gómez-Coca, L. E. Kapinos, A. Holý, R. A. Vilaplana, F. González-Vílchez and H. Sigel, *J. Chem. Soc., Dalton Trans.*, 2077-2084 (2000).

12. (a) H. Sigel, *Chem. Soc. Rev.*, **22**, 255-267 (1993). (b) H. Sigel, S. S. Massoud and N. A. Corfù, *J. Am. Chem. Soc.*, **116**, 2958-2971 (1994).

13. E. M. Bianchi, S. A. A. Sajadi, B. Song and H. Sigel, *Chem. Eur. J.*, **9**, 881-892 (2003).

14. C. A. Blindauer, A. H. Emwas, A. Holý, H. Dvořáková, E. Sletten and H. Sigel, *Chem. Eur. J.*, **3**, 1526-1536 (1997).

15. K. Aoki, *Met. Ions Biol. Syst.*, **32**, 91-134 (1996).

16. A. Holý, H. Dvořáková, J. Jindřich, M. Masojídková, M. Buděšínský, J. Balzarini, G. Andrei and E. De Clercq, *J. Med. Chem.*, **39**, 4073-4088 (1996).

17. (a) H. Dvořáková, A. Holý, M. Masojídková, I. Votruba, J. Balzarini, R. Snoeck and E. De Clercq, *Collect. Czech. Chem. Commun.*, **58**, Special issue, 253-255 (1993). (b) P. Franchetti, G. Abu Sheikha, L. Cappellacci, L. Messini, M. Grifantini, A. G. Loi, A. De Montis, M. G. Spiga and P. La Colla, *Nucleosides & Nucleotides*, **13**, 1707-1719 (1994).
18. (a) R. B. Martin, *Inorg. Chim. Acta*, **339**, 27-33 (2002). (b) H. Sigel and D. B. McCormick, *Acc. Chem. Res.*, **3**, 201-208 (1970).
19. R. B. Gómez-Coca, L. E. Kapinos, A. Holý, R. A. Vilaplana, F. González-Vílchez and H. Sigel, *Metal Based Drugs*, **7**, 313-324 (2000).
20. H. Sigel, A. D. Zuberbühler and O. Yamauchi, *Anal. Chim. Acta*, **255**, 63-72 (1991).
21. H. M. Irving, M. G. Miles and L. D. Pettit, *Anal. Chim. Acta*, **38**, 475-488 (1967).
22. C. A. Blindauer, T. I. Sjåstad, A. Holý, E. Sletten and H. Sigel, *J. Chem. Soc., Dalton Trans.*, 3661-3671 (1999).
23. H. Sigel, R. Griesser and B. Prijs, *Z. Naturforsch.*, **27b**, 353-364 (1972).
24. O. Yamauchi, A. Odani, H. Masuda and H. Sigel, *Met. Ions Biol. Syst.*, **32**, 135-205 (1996).
25. W. S. Sheldrick and G. Heeb, *Inorg. Chim. Acta*, **190**, 241-248 (1991).
26. C. A., Blindauer, A. Holý, H. Dvořáková and H. Sigel, *J. Chem. Soc., Perkin Trans. 2*, 2353-2363 (1997).
27. (a) G. Kampf, L. E. Kapinos, R. Griesser, B. Lippert and H. Sigel, *J. Chem. Soc., Perkin Trans. 2*, 1320-1327 (2002). (b) C. Meiser, B. Song, E. Freisinger, M. Peilert, H. Sigel and B. Lippert, *Chem. Eur. J.*, **3**, 388-398 (1997).
28. M. J. Sánchez-Moreno, R. B. Gómez-Coca, A. Fernández-Botello, J. Ochocki , A. Kotynski, R. Griesser and H. Sigel, *Organ. Biomol. Chem.*, **1**, 1819-1826 (2003).
29. A. Albert, *J. Chem. Soc. (C)*, 152-160 (1969).
30. C. A. Blindauer, A. Holý and H. Sigel, *Collect. Czech. Chem. Commun.*, **64**, 613-632 (1999).
31. (a) H. Sigel, C. P. Da Costa, B. Song, P. Carloni and F. Gregáň, *J. Am. Chem. Soc.*, **121**, 6248-6257 (1999). (b) C. P. Da Costa and H. Sigel, *J. Biol. Inorg. Chem.*, **4**, 508-514 (1999).
32. (a) L. E. Kapinos, A. Holý, J. Günter and H. Sigel, *Inorg. Chem.*, **40**, 2500-2508 (2001). (b) L. E. Kapinos, B. Song and H. Sigel, *Z. Naturforsch.*, **53b**, 903-908 (1998).
33. H. Sigel, *Eur. J. Biochem.*, **3**, 530-537 (1968).
34. (a) H. Sigel and B. Lippert, *Pure Appl. Chem.*, **70**, 845-854 (1998). (b) B. Song, J. Zhao, R. Griesser, C. Meiser, H. Sigel and B. Lippert, *Chem. Eur. J.*, **5**, 2374-2387 (1999).
35. R. Griesser, G. Kampf, L. E. Kapinos, S. Komeda, B. Lippert, J. Reedijk and H. Sigel, *Inorg. Chem.*, **42**, 32-41 (2003).
36. (a) C. A. Blindauer A. Holý, H. Dvořáková and H. Sigel, *J. Biol. Inorg. Chem.*, **3**, 423-433 (1998). (b) M. S. Lüth, L. E. Kapinos, B. Song, B. Lippert and H. Sigel, *J. Chem. Soc., Dalton Trans.*, 357-365 (1999).
37. R. B. Martin and H. Sigel, *Comments Inorg. Chem.*, **6**, 285-314 (1988).
38. S. S. Massoud and H. Sigel, *Inorg. Chem.*, **27**, 1447-1453 (1988).
39. H. Sigel and B. Song, *Met. Ions Biol. Syst.*, **32**, 135-205 (1996).

40. H. Sigel and L. E. Kapinos, *Coord. Chem. Rev.*, **200-202**, 563-594 (2000).

41. (a) S. S. Massoud and H. Sigel, *Eur. J. Biochem.*, **179**, 451-458 (1989). (b) A. Marzotto, A. Ciccarese, D. A. Clemente and G. Valle, *J. Chem. Soc., Dalton Trans.*, 1461-1468 (1995). (c) M. A. Billadeau and H. Morrison, *Met. Ions Biol. Syst.*, **33**, 269-296 (1996); see p. 279. (d) W. Wirth, J. Blotevogel-Baltronat, U. Kleinkes and W. S. Sheldrick, *Inorg. Chim. Acta*, **339**, 14-26 (2002). (e) E. Bugella-Altamirano, D. Choquesillo-Lazarte, J. M. González-Pérez, M. J. Sánchez-Moreno, R. Marín-Sánchez, J. D. Martín-Ramos, B. Covelo, R. Carballo, A. Castiñeiras and J. Niclós-Gutierrez, *Inorg. Chim. Acta*, **339**, 160-170 (2002).

42. R.B. Gómez-Coca, L.E. Kapinos, A. Holy, R.A. Vilaplana, F. González-Vílchez and H. Sigel, *J. Biol. Inorg. Chem.*, **9**, in press (2004).

